# The Etiology of Diphtheria

**Published:** 1896-07-18

**Authors:** 


### Medical Progress and Hospital Clinics.

[*The Editor will be glad to receive offers of co-operation and contributions from members of the profession. All letters should be addressed to* The Editor, at the Office, 428, Strand, London, W.C.]

### THE ETIOLOGY OF DIPHTHERIA.

Some important observations have recently been made at the North-Eastern Hospital of the Asylums Board in regard to the relation between diphtheria and the bacilli found in that disease.

It has long been known that in many cases the bacilli of diphtheria can be found in the throat by bacteriological investigation, although the patients may present no evidence of being affected by the disease. But at this hospital it was found that such persons ran great risks if allowed to associate with those suffering from clinical diphtheria. Several patients who were removed from the general to the diphtheria wards, not because they were suffering from that disease but because they had the bacilli in their throats, ending by catching true diphtheria and in some cases dying of it. Clearly these people, for all that they carried the bacilli in their throats, were by no means immune to clinical diphtheria, for they caught it when exposed to it, and the previous innocuousness of the bacilli must have been due to the absence of some condition essential to their virulence. What, then, is this condition?

The problem is a most complex one, and it will probably be long before a completely satisfactory solution is discovered. Nevertheless, it may not be out of place to suggest that the most hopeful line of research lies in the investigation of the nature of the circumstances under which diphtheria most readily occurs, rather than in hunting out further details regarding the bacillus which we already know. There are two sets of facts which just now seem peculiarly suggestive. On the one hand we have the well-known observations on concurrent infection, according to which microbes, which when isolated may be innocuous, become actively virulent when grown in conjunction with other forms, which themselves also are harmless when alone; and, on the other, we have a whole series of observations, made in many cases before the discovery of the bacillus, as to the conditions under which diphtheria tends to become prevalent, and especially as to its association with forms of sore throat which themselves seem to be more or less infectious.

In view of what we know as to the frequent latency of diphtheria, it may be accepted that when this disease manifests itself after exposure to foul emanations, which are known to produce sore throats, these emanations must not be regarded as the cause of the diphtheria, but as the origin of a condition of throat in which the already present bacilli are able to manifest their full morbific virulence.

Much has been said about the "progressive development of the property of infectiveness," and about an epidemic gradually "working up" from one of ordinary sore throat to one of true diphtheria; but, while fully accepting the correctness of such observations, it may be said that such a "progressive development" may merely mean a progressive prevalence of that sort of sore throat in the presence of which diphtheria can most readily become virulent.

What we want now to find out is, what there is common to the varied conditions amid which diphtheria most frequently arises—damp soils, cold and variable climates, deficient drainage, foul emanations, crowding in schools, crowding in hospitals, "common sore throats," the condition of throat left by scarlet fever, and that something, depending on climatic conditions, which in these isles makes the period between September and the end of the year the season for diphtheria's greatest prevalence. The presumption is strong that this common condition is another microbe, producing another condition of throat, more or less infectious; and that while either the diphtheria microbe or this unknown microbe can be transmitted independently without producing very evil effects, their coincidence produces what is known as clinical diphtheria. This would explain the very different behaviour of clinical and bacteriological diphtheria. In the one case, both infections being concurrently transmitted, the full disease would be developed, whereas around these cases would be numbers in which only one or other form had taken root, the cases of bacteriological diphtheria being due to the diphtheria bacillus alone, while the crowd of sore throats which precede and surround epidemics of diphtheria would be due to the so far unknown (microbic?) cause.

The investigation of the etiology of diphtheria must not, however, be left altogether to the bacteriologist. One important clue is given by the observations at the Asylums Board hospitals, viz., as to the effect of the aggregation of many cases of sore throat in leading to outbreaks of diphtheria; and it is by no means improbable that this may be the essential factor in what is called "school influence." This clue should be followed up by medical officers of health in their investigations into epidemics of diphtheria due to milk. Too often in such inquiries they have been content to convict the milk. But if we are to accept the official view, and take it as proved that milk from certain cows is capable of producing diphtheria, we must go further, and when we find the infection sown evenly over a district, we must next proceed to inquire what sort of people it selects, and what sort it passes by.

The tendency which diphtheria has shown of recent years to invade large towns has drawn forth various explanations, more or less unsatisfactory and conflicting. But if we consider certain social changes which have taken place during the same period the true explanation may be nearer than we think. Granting that diphtheria springs from a disease of the cow and is distributed by milk, we at once perceive that, while the railways have brought country milk and its corollary country diphtheria in great abundance to the towns, the complaint has arisen on every hand that the common people in the country can get no milk. On the other hand, accepting the hint derived from the hospitals as to the effect of overcrowding, and the strong evidence as to "school influence," which is also but a form of promiscuous overcrowding, we are reminded at once that the modern tendency of our large towns is towards over-crowded tenements, while country distress tends to the emptying of country homes.

The investigation of the causes of diphtheria must, then, not lie altogether in the laboratory. There is a vast field open also for clinical observers, and for sanitary and statistical experts.

